# Growth analysis and yield of two varieties of groundnut (*Arachis hypogaea* L.) as influenced by different weed control methods

**DOI:** 10.1007/s40502-015-0151-x

**Published:** 2015-03-27

**Authors:** Bolaji U. Olayinka, Emmanuel O. Etejere

**Affiliations:** Department of Plant Biology, P.M.B. 1515, University of Ilorin, Ilorin, Nigeria

**Keywords:** Leaf area index, Net assimilation rate, Relative growth rate, Straw mulching, Yield

## Abstract

Field trials were carried out to evaluate the effects of seven weed management strategies on the growth and yield of two groundnut varieties (Samnut 10 and MK 373) for two successive seasons (2010–2011). The experimental layout was a split plot complete randomized block design with three replications. The two groundnut varieties showed identical pattern of results for leaf area index, dry matter accumulation, relative growth rate, net assimilation rate and crop growth rate as well as yield. All the weed control treatments significantly enhanced the growth and yield compared with the weedy check. The weed free check had the highest growth but the highest yield was recorded from rice straw mulch at 0.1 m depth + one hand weeding at 6 weeks after sowing (WAS) due to increase in number of matured pods per plant, seed weight per plant and 100-seed weight. The results showed that rice straw mulch at 0.1 m depth + one hand weeding at 6 WAS was better agronomical practice for enhancing growth and yield of groundnut. This enhancement could be as a result of its positive influence on physiological parameters such as leaf area index, dry matter accumulation, relative growth rate, net assimilation rate and crop growth rate. Its use is also ecofriendly as it limits the need for synthetic herbicide.

## Introduction

Groundnut is a major oil seed crop grown in tropics and sub-tropic parts of the world. Nigeria is the fourth largest producer of groundnut after China, India and USA (USDA 2009). In United States of America groundnut yield is as high as 3000 kg ha^−1^, while the average yield in tropical Africa is about 800 kg ha^−1^, which is traceable to weed infestation (Akobundu 1987). In Nigeria, groundnut yield loss could be as high as 51 % (Etejere et al. 2013). This is because in groundnut, less crop canopy during the first 6 weeks of growth favours strong competition with weeds causing significant reduction in yield (Akobundu 1987).

The degree of damage caused by the weeds has been found to be a function of their leaf area index (LAI) as compared to the crop they are competing with (El-Naim and Ahmed 2010). Vijay Kumar (1992) found that groundnut growth in the absence of weeds attained maximum LAI. LAI, therefore, is an important determinant of dry matter accumulation and grain yield, and it can be adjusted by various agronomical practices (Rahman et al. 1994).

Nakaseko et al. (1979) had observed that high crop productivity can be achieved by exploring the pattern of dry matter accumulation and partitioning, which helps in adjusting proper crop management practices. However, studies of dry matter accumulation and partitioning in relation to yield of groundnut under organic mulch treatment in comparison to hand weeding and chemical weed control are very limited. By exploring the pattern of dry matter partitioning in relation to LAI, the present investigation was carried out to compare and evaluate the effects of rice straw mulch either sole or integrated with one handing weeding with other weed control methods on physiological performance of two groundnut varieties.

## Materials and methods

The field trials reported here was carried out at Lafiagi—a Guinea savanna zone of Nigeria (latitude 8°50′N and longitude 5°25′E) in 2010 and 2011. The soil of the experimental field was sandy-loam, with low organic matter (0.74 %), moderately high in available nitrogen (0.25 %), and slightly acidic pH (6.35). The total rainfall received during the cropping seasons at the site was less in 2010 (844.6 mm) than 2011 (1030.3 mm). The mean relative humidity and average temperature were 35.7 °C and 80.9 %; 32.4 °C and 84.1 % in 2010 and 2011, respectively. Predominant weed species were *Brachiaria deflexa, Cleome viscosa, Cochlospermum planchoni, Dactyloctenium aegyptium and Daniellia oliveri.*

### Description of the varieties and field operation

Two varieties of groundnut Samnut 10 and MK 373 were used for the study. Samnut 10 is erect with small pod containing 1–2 seeds. It matures between 98–105 days. MK 373 is decumbent type. Pod is large with moderate constriction. Pod contains 2–4 seeds. It matures earlier than Samnut 10 (84–92 days). The seeds of the two varieties used in this study were obtained from College of Agriculture, Mokwa, Niger State. Before sowing seeds were treated with Seedrex (33 % permethrin + 15 % carbonderzine + 12 % chlorothalonil) at the rate of 10 g per 4 kg of seeds to prevent soil borne diseases.

### Experimental layout and treatment details

The experiment was laid out in split plots design, with varieties in the main plot and the weed control treatments as the subplot. The various weed control treatments investigated were arranged to fit a complete randomized block design with three replications. Plot size was 3 m × 3 m consisting of four rows spaced 0.4 m apart with 0.1 m inter-crop spacing. Data on weeds and crop were collected within the two inner rows of 4.8 m^2^ leaving the outer rows as buffer. The treatments consisted of seven weed control strategies: T_1_: Pendimethalin at 1.5 l ha^−1^ as pre-emergence (water at 600 l ha^−1^ was used as carrier after proper calibration with the aid of Knapsack sprayer of 20 liters capacity) T_2_: Pendimethalin at 1.5 l ha^−1^ as pre emergency + one hand weeding at 6 weeks after sowing (WAS), T_3_: Rice straw mulch at 0.1 m depth (straw was laid in the inter-row) T_4_: Rice straw mulch at 0.1 m depth + one hand weeding at 6 WAS, T_5_: Two hand weeding at 3 and 6 WAS, T_6_: Weed free check (continuous weeding was done at interval of 2 weeks) and T_7_: Weedy check (plots were infested with weeds throughout the period of the experiment.

### Data collection

Weed count was achieved by placing three quadrates of 1 m^2^ at random in each subplot within the weed management zone of 4.8 m^2^. Weeds rooted within this zone in each treatment were counted at harvest. The harvested weeds were oven dried at 80 °C to a constant weight to determine the weed biomass. The weed control efficiency and weed index were calculated following the formula of Gill and Kumar (1966).

Plant samples were harvested at interval of 2 weeks within the weed management zone. The above-ground portion of the harvested crop was first washed in running water and segmented into leaves and stems. Thereafter, these plant parts were oven dried at 80 °C to constant weight. The dry weight of different plant parts were then measured using Electronic 101 Balance with precision of 0.1 g. LAI was estimated as (LAI = surface area of sampled leaf/ground area occupied by the sampled plants). Physiological parameters such as relative growth rate (RGR), net assimilation rate (NAR) and crop growth rate (CGR) were estimated according to Gardner (1985). In each experimental plot, data on seed and pod yield were recorded on 10 randomly selected plants harvested within weed management zone of 4.8 m^2^ after drying to 12 % moisture. Harvest index and yield attributes such as number of matured pod per plant, seed weight per plant and 100-seed weight were also determined.

### Data analysis

Data recorded in 2010 and 2011 cropping seasons were pooled together on account of non-significant interaction between years, treatment and variety. The data were then subjected to Univariate Analysis of Variance. Treatment means were separated using least significant difference (LSD) at 0.01 probability level.

## Results and discussion

### Weed control effect

Various weed control methods significantly (p ≤ 0.01) influenced weed biomass, weed control efficiency and weed index (Table 1).Weed biomass was significantly higher in weedy check in Samnut 10 (662.1 g m^−2^) and MK 373 (686.26 g m^−2^) as compared to other weed control treatments. Significantly lowest weed biomass was recorded in weed free check in both Samnut 10 and MK 373 with values of 68.51 and 71.71 kg ha^−1^, respectively. In both varieties, sole pendimethalin at 1.5 l ha^−1^ and rice straw mulch had significantly higher weed biomasses as compared to rice straw mulch + one hand weed at 6 WAS, pendimethalin at 1.5 l ha^−1^ + one hand weeding at 6 WAS and two hand weeding at 3 and 6 WAS (Table 1). The results of weed control efficiency were significantly higher in weed free check (94–95 %), and followed an inverse relationship with weed biomass (Table 1). The weed index followed the same trend as weed biomass (Table 1). The results showed that all the weed control treatments were effective in reducing weed biomass compared to the weedy check. However, sole application of rice straw mulch and pendimethalin at 1.5 l ha^−1^ were not very effective in checking weed growth, as evident from the higher weed index and lower weed control efficiency as compared to other weed control treatments. Straw mulch is known to suppress weeds to some extent (Singh 2009). With regard to sole application of pendimethalin at 1.5 l ha^−1^, the results corroborates the earlier studies of Olorunmaiye and Olorunmaiye (2009), who reported that pre-emergence herbicide treatments without supplementary hoe-weeding could not provide season long weed control because of their short persistence.

**Table 1:** Effect of different weed control methods on weed biomass, weed control efficiency, weed index and leaf area index in Samnut 10 and MK 373 (pooled data of cropping seasons 2010 and 2011)

Variety	Treatment	Weed biomass (g m^−2^)	Weed control efficiency (%)	Weed index	Leaf area index (%) Week after sowing		
					8	10	12
Samnut 10	T_1_	248.22^c^	63.54^c^	13.79^b^	2.93^a^	3.20^ab^	2.60^ab^
	T_2_	128.34^d^	77.75^b^	−2.03^c^	3.43^a^	3.93^ab^	2.90^ab^
	T_3_	335.79^b^	46.92^d^	18.58^b^	3.20^a^	3.30^ab^	2.63^ab^
	T_4_	122.54^e^	81.11^a^	−20.47^e^	3.97^a^	4.63^a^	3.73^ab^
	T_5_	132.26^d^	82.58^a^	−12.54^d^	3.63^a^	4.13^ab^	3.60^ab^
	T_6_	32.88^f^	94.94^a^	0.000^c^	3.83^a^	4.93^a^	3.93^a^
	T_7_	662.11^a^	0000^e^	47.03^a^	2.47^a^	2.63^b^	1.93^b^
	Mean	237.45	63.83	44.36	3.35	3.82	3.05
MK 373	T_1_	258.16^c^	61.09^c^	11.30^bc^	3.93^a^	3.17^bc^	2.63^a^
	T_2_	151.36^d^	79.83^b^	−18.17^cd^	4.50^a^	3.83^ab^	3.53^a^
	T_3_	370.22^b^	46.94^d^	18.17^b^	4.00^a^	3.33^abc^	2.87^a^
	T_4_	133.67^e^	78.84^b^	−14.10^d^	4.63^a^	4.10^a^	3.83^a^
	T_5_	108.16^f^	80.78^b^	−6.78 ^cd^	4.37^a^	3.90^ab^	3.63^a^
	T_6_	34.42^g^	95.11^a^	0000^bcd^	4.70^a^	4.10^a^	3.97^a^
	T_7_	686.26^a^	0000^e^	40.53^a^	3.43^a^	2.87^c^	2.47^a^
	Mean	248.89	63.17	30.95	4.22	3.62	3.28
	Grand mean	243.17	63.50	37.66	3.79	3.72	3.17
LSD (p < 0.01)	Variety	36.85	11.98	99.43	0.94	0.74	4.00
	Treatment	16.32	25.80	10.74	0.63	0.95	0.56
	V × T	23.60	12.23	43.33	0.57	0.57	0.21

Within column means followed by the same superscripts are not significantly different at p ≤ 0.01

T_1_: Pendimethalin at 1.5 l ha^−1^, T_2_: Pendimethalin at 1.5 l ha^−1^ + one hand weeding at 6 WAS, T_3_: Rice straw mulch, T_4_: Rice straw mulch + one hand weeding at 6 WAS, T_5_: Two hand weeding at 3 and 6 WAS, T_6_: Weed free check (positive control), T_7_: Weedy check (negative control)

The reduction in weed biomass by rice straw mulch + one hand weeding at 6 WAS, two hand weeding at 3 and 6 WAS and pendimethalin at 1.5 l ha^−1^ + one hand weeding at 6 WAS was because these treatments prevented further fresh flush of weeds besides suppressing the already emerged ones. Evidence in support of this was observed in weed control efficiency, which was as high as 72–82 %, when either two hand weeding were carried out at 3 and 6 WAS or rice straw mulch or pendimethalin were integrated with one hand weeding at 6 WAS compared to the sole pendimethalin and sole rice straw mulch (46–63 %). Similar results have been reported by Agasimani et al. (2010) and El-Naim et al. (2011), where integrated weed control methods or two hand weeding had been practiced in groundnut.

### Leaf area index (LAI)

In both varieties, LAI was significantly (p ≤ 0.01) affected at different growth periods except at 8 WAS (Table 1). In case of Samnut 10, highest LAI was recorded at 10 WAS in all the treatments, whereas in MK 373 LAI was higher at 8 WAS (Table 1). During these periods, the weed free check showed significantly higher LAI values of 4.93 and 4.70 in Samnut 10 and MK 373, respectively (Table 2). These were followed by rice straw mulch + one hand weeding at 6 WAS with value of 4.63 in Samnut 10 and 4.65 in MK 373 (Table 1). During these growth periods, lowest LAI was recorded under the weedy check as compared to other weed control treatments. Treatments such as two hand weeding at 3 and 6 WAS and pendimethalin at 1.5 l ha^−1^ + one hand weeding at 6 WAS had LAI values that were statistically similar to those of rice straw mulch + one hand weeding either at 6, 8, 10 and 12 WAS. Sole pendimethalin and sole rice straw mulch showed lower LAI values as compared to other weed control treatments. As observed in this study, treatments with higher weed control efficiency (rice straw mulch + one hand weeding at 6 WAS, two hand weeding at 3 and 6 WAS and pendimethalin at 1.5 l ha^−1^ + one hand weeding at 6 WAS) had higher LAI as compared to those with low weed control efficiency (sole rice straw mulch and sole pendimethalin). Effective weed control has been observed to enhance LAI in groundnut (Kumar 2009). Singh (2003) observed that in Sourashtra region of India, LAI of bunch varieties of groundnut might be 1.7 at 60 days after planting (DAP), and might increased to 4.0 at 90 DAP. McCloud (1974) observed that at 64 DAP, LAI of groundnut might reach 3.0, which at maturity (137 DAP) might reduced to 1.7. In this present study, both varieties, reached highest LAI values of 4.63–4.93, which reduced to 1.93–2.47 at maturity. The reduction in LAI at 12 WAS in Samnut 10 and 10–12 WAS in MK 373 was due to pest attack (Banik et al. 2009).

**Table 2:** Effect of different weed control methods on above-ground dry weight and relative growth rate at different crop growth stages in Samnut 10 and MK 373 (pooled data of cropping seasons 2010 and 2011)

Variety	Treatment	Week after sowing					
		Above-ground dry weight (g)			Relative growth rate (g g^−1 ^ day^−1^)		
		8	10	12	6–8	8–10	10–12
Samnut 10	T_1_	18.93^d^	23.07^d^	18.00^d^	0.49^a^	0.68^a^	0.11^c^
	T_2_	22.50^c^	31.77^c^	25.27^c^	0.51^a^	0.83^a^	0.28^b^
	T_3_	20.00^d^	23.17^d^	19.07^d^	0.57^a^	0.68^a^	0.09^c^
	T_4_	29.17^b^	36.43^b^	28.93^a^	0.83^a^	0.89^a^	0.11^c^
	T_5_	29.53^ab^	36.17^b^	27.33^b^	0.72^a^	0.81^a^	0.13^c^
	T_6_	31.33^a^	38.73^a^	29.47^a^	0.72^a^	0.92^a^	0.58^a^
	T_7_	9.77^e^	15.77^e^	12.27^e^	0.46^a^	0.57^a^	0.12^c^
	Mean	23.03	29.30	22.90	0.62	0.77	0.20
MK 373	T_1_	22.27^c^	31.33^e^	31.47^c^	0.67^ab^	0.15^ab^	0.01^b^
	T_2_	32.23^a^	35.93^c^	32.33^c^	0.89^ab^	0.04^b^	0.13^a^
	T_3_	28.37^b^	33.30^d^	29.03^d^	0.60^ab^	0.06^ab^	0.10^ab^
	T_4_	34.47^a^	42.07^b^	45.13^a^	0.89^ab^	0.10^ab^	0.04^ab^
	T_5_	33.90^a^	46.57^a^	40.43^b^	0.81^ab^	0.15^ab^	0.08^ab^
	T_6_	34.80^a^	42.87^b^	46.40^a^	0.93^a^	0.11^ab^	0.04^ab^
	T_7_	14.53^d^	22.40^f^	20.47^e^	0.49^b^	0.18^a^	0.01^b^
	Mean	28.65	36.35	35.04	0.75	0.11	0.06
	Grand mean	25.84	32.83	28.97	0.69	0.44	0.04
LSD (p ≤ 0.01)	Variety	5.56	4.30	4.00	0.13	0.06	0.01
	Treatment	1.44	0.93	1.02	0.50	0.06	0.03
	V × T	0.66	0.19	0.31	0.02	0.02	0.03

Within column means followed by the same superscripts are not significantly different at p ≤ 0.01

T_1_: Pendimethalin at 1.5 l ha^−1^, T_2_: Pendimethalin at 1.5 l ha^−1^ + one hand weeding at 6 WAS, T_3_: Rice straw mulch, T_4_: Rice straw mulch + one hand weeding at 6 WAS, T_5_: Two hand weeding at 3 and 6 WAS, T_6_: Weed free check (positive control), T_7_: Weedy check (negative control)

### Dry matter production

Dry matter accumulation at 8, 10 and 12 WAS in the two varieties differed significantly (p ≤ 0.01) due to different weed control treatments (Table 2). Highest dry matter production was recorded in the weed free check Among the weed control treatments, rice straw mulch + one hand weeding at 6 recorded highest dry matter accumulation, followed by two hand weeding at 3 and 6 WAS and Pendimethalin at 1.5 l ha^−1^ + one hand weeding at 6 WAS, sole rice straw mulch and pendimethalin at 1.5 l ha^−1^. The significantly lower dry matter was recorded in weedy check over all other treatments. Variation in dry matter accumulation could be due to differences in the leaf area production and LAI. Similarly, reduction in weed competition for limited resources in treatments that had higher dry matter accumulation could have stimulated stronger carbohydrates sinks via photosynthesis. Highest dry matter accumulation in rice straw mulch + one hand weeding at 6 WAS could be linked to modification of growing environment for enhanced productivity. In this study, LAI showed positive correlation with the dry matter accumulation (r^2^ = 0.690). Agasimani et al. (2010) observed higher dry matter production in treatments, where integrated weed management was practiced in groundnut-wheat cropping system in Northern Karnataka.

### Relative growth rate (RGR)

Relative growth rate was highest between 6 and 8 WAS in MK 373 and 8–10 WAS in Samnut 10 and declined with advent of time (Table 2). All the weed control treatments had higher RGR as compared to the weedy check. This showed that growth of groundnut in weedy check was grossly affected by weed infestation. Better, RGR observed between 6 and 8 and 8–10 WAS in both the varieties in treatments such as weed free check, rice straw mulch + one hand weeding at 6 WAS, two hand weeding at 3 and 6 WAS and pendimethalin at 1.5 l ha^−1^ + one hand weeding at 6 WAS could be attributed to effective weed control and low weed biomass, which enabled plant receiving these treatments higher photosynthetic rate. The lower RGR in sole rice straw mulch and sole pendimethalin, though higher than the weedy check during these periods, could be due to the inability of these treatments to offer season-long weed control. The decline in RGR towards physiological maturity could be due to leaf shedding, shadow of upper leaves over the lower leaves which reduce the photosynthetic capacity of the lower leaves and finally loss of leaves due to pest attack (Banik et al. 2009).

### Net assimilation rate (NAR)

Net assimilation rate followed the same trend as RGR (Table 3). At peak periods, between −8 and 8–10 WAS in MK 373 and Samnut 10, respectively, NAR, was highest in weed free plots or plot raised under rice straw mulch + one hand weeding at 6 WAS, followed by two hand weeding at 3 and 6 WAS and pendimethalin at 1.5 l ha^−1^ + one hand weeding at 6 WAS, sole rice straw mulch and pendimethalin at 1.5 l ha^−1^ (Table 3). Lowest NAR was recorded in weedy check. Greater NAR in treatments that showed effective weed control could be due to higher photosynthetic rate on account of reduced weed competition for limited resources such as water, light, nutrient and space. Singh (2004) observed that photosynthetic rate of leaves decrease as the relative water content and water potential decreases in groundnut. This also explains the decrease in NAR in treatments such as sole pendimethalin at 1.5 l ha^−1^, sole rice straw mulch and the weedy check, where high weed biomass affected the water potential and consequently reduced the photosynthetic efficiency. The decrease in NAR in groundnut towards physiological maturity may be linked to mutual shading, increase in number of old leaves with low photosynthetic efficiency and loss of leaves due to pest attack (Banik et al. 2009).

**Table 3:** Effect of different weed control methods on net assimilation rate and crop growth rate at different crop growth stages in Samnut 10 and MK 373 (pooled data of cropping seasons 2010 and 2011)

Variety	Treatment	Week after sowing (WAS)					
		Net assimilation rate (g m^−2^ day^−1^)			Crop growth rate (g m^−2^ day^−1^)		
		6–8	8–10	10–12	6–8	8–10	10–12
Samnut 10	T_1_	0.006^a^	0.010^b^	0.005^c^	1.73^d^	3.10^e^	1.31^e^
	T_2_	0.023^a^	0.040^a^	0.008^c^	5.17^c^	5.53^c^	2.47^d^
	T_3_	0.005^a^	0.014^b^	0.005^c^	1.93^d^	4.87^d^	1.43^e^
	T_4_	0.025^a^	0.046^a^	0.039^b^	7.40^ab^	8.83^a^	6.40^a^
	T_5_	0.025^a^	0.044^a^	0.058^a^	6.50^b^	6.83^a^	4.17^c^
	T_6_	0.025^a^	0.048^a^	0.010^c^	8.03^a^	8.87^a^	5.79^a^
	T_7_	0.008^a^	0.008^b^	0.005^c^	2.20^d^	2.33^f^	1.13^e^
	Mean	0.017	0.030	0.019	4.71	5.77	3.24
MK 373	T_1_	0.019^ab^	0.010^b^	0.001^b^	5.53^f^	3.47^de^	2.13^c^
	T_2_	0.031^abc^	0.019^a^	0.005^b^	7.23^d^	6.43^b^	4.27^b^
	T_3_	0.017^bc^	0.009^a^	0.072^a^	5.93^e^	3.22^e^	2.33^c^
	T_4_	0.042^ab^	0.008^b^	0.004^b^	9.17^b^	6.17^b^	1.90^c^
	T_5_	0.041^ab^	0.009^b^	0.007^b^	7.57^c^	4.27^c^	4.80^ab^
	T_6_	0.046^a^	0.008^b^	0.007^b^	10.57^a^	7.00^a^	5.30^a^
	T_7_	0.012^c^	0.011^b^	0.002^b^	4.37^g^	3.83^cd^	2.30^c^
	Mean	0.030	0.011	0.010	7.20	4.91	3.30
	Grand mean	0.024	0.021	0.015	5.96	5.34	3.27
LSD (p ≤ 0.01)	Variety	0.0022	0.00016	0.0022	0.14	0.57	0.39
	Treatment	0.020	0.021	0.020	0.27	0.64	0.28
	V × T	0.0001	0.00005	0.0001	0.12	0.01	0.04

Within column means followed by the same superscripts are not significantly different at p ≤ 0.01

T_1_: Pendimethalin at 1.5 l ha^−1^, T_2_: Pendimethalin at 1.5 l ha^−1^ + one hand weeding at 6 WAS, T_3_: Rice straw mulch, T_4_: Rice straw mulch + one hand weeding at 6 WAS, T_5_: Two hand weeding at 3 and 6 WAS, T_6_: Weed free check (positive control), T_7_: Weedy check (negative control)

### Crop growth rate (CGR)

Crop growth rate was significantly (p ≤ 0.01) affected at different growth periods by weed control treatments (Table 3). Apart from weed free check, from 6–8 to 10–12 WAS, CGR was highest in rice straw mulch + one handing at 6 WAS, followed by two hand weeding at 3 and 6 WAS, pendimethalin at 1.5 l ha^−1^ + one hand weeding, sole rice straw and sole pendimethalin at 1.5 l ha^−1^. Lowest CGR was recorded in weedy check at all growth periods. The highest CGR in either weed free check or rice straw mulch + one hand weeding at 6 WAS might be due to less weed competition for natural resources such as water, light, nutrients and space for crop growth. Also higher level of LAI and NAR could account for higher CGR recorded under these treatments (Augadi et al. 1990). Varietal differences showed that MK 373 had higher CGR than Samnut 10 and this could be due to more dry matter accumulation in former than latter. Singh and Joshi (1993) have reported Virginia runner cultivar had higher pod number and yield than the erect type due to more dry matter, N-accumulation and higher CGR. The decrease in CGR after 6–8 and 8–10 WAS in MK 373 and Samnut 10, respectively could be due to loss of leaves on account of pest attack and mutual shading of leaves, which was higher in treatments where more leaves were produced.

### Yield components and yield

Weed control methods significantly affected yield components and yield in both the varieties (Table 4). In both varieties, highest number of matured pods per plant, seed weight per plant, 100-seed weight, pod yield, seed yield and harvest index were recorded in rice straw mulch + one hand weeding at 6 WAS over all other treatments (Table 4). Lowest yield components and yield were recorded in weedy check. The highest yield recorded in rice straw mulch + one hand weeding at 6 WAS might be due to reduced weed competition for limited resources resulting in increase in 100-seed weight, number of matured pods per plant, seed weight per plants compared to other treatments. Additionally, higher moisture content and the decomposition of the mulch could have also contributed to increase in supply of nutrients for the overall increase in yield in this treatment. The results supported the opinion of Singh and Joshi (1993), where higher pod yield is attributed to better N accumulation, higher dry matter and CGR. Similarly, groundnut yields have been found to be enhanced, where integrated weed control methods were practiced (Kumar 2009; Jat et al. 2011). In the present study, seed yield correlated positively with all the physiological growth parameters (r^2^ = 0.69–0.95) evaluated.

**Table 4:** Effect of different weed control methods on yield components and yield in Samnut 10 and MK 37 (pooled data of cropping seasons 2010 and 2011)

Variety	Treatment	Number of matured pod per plant	Seed weight (g plant^−1^)	100-seed weight (g)	Pod yield (g m^−2^)	Seed yield (g m^−2^)	Harvest index
Samnut 10	T_1_	24.67^d^	10.67^d^	37.73^b^	397.13^c^	280.29^e^	0.25^a^
	T_2_	32.33^c^	15.47^c^	38.58^b^	475.56^b^	349.39^a^	0.32^a^
	T_3_	23.17^e^	10.53^d^	38.10^b^	386.76^d^	278.36^f^	0.26^a^
	T_4_	45.33^a^	22.37^a^	40.18^a^	542.78^a^	384.84^a^	0.39^a^
	T_5_	44.17^b^	21.25^b^	40.12^a^	534.77^a^	376.89^b^	0.36^a^
	T_6_	32.67^c^	15.78^c^	38.48^a^	529.63^a^	353.19^c^	0.30^a^
	T_7_	13.50^f^	6.17^e^	35.42^c^	264.42^e^	172.24^g^	0.24^a^
	Mean	30.50	14.59	38.43	447.22	313.61	0.30
MK 373	T_1_	28.17^b^	15.23^b^	40.27^c^	446.19^d^	305.19^e^	0.26^a^
	T_2_	30.00^b^	20.60^b^	42.63^ab^	533.57^c^	388.51^d^	0.35^a^
	T_3_	21.00^c^	12.55^c^	40.77^bc^	416.43^e^	292.58^f^	0.32^a^
	T_4_	35.67^a^	26.02^a^	44.38^a^	637.15^a^	466.41^a^	0.36^a^
	T_5_	33.33^a^	24.08^a^	44.12^a^	569.86^b^	424.85^b^	0.34^a^
	T_6_	33.33^a^	21.58^a^	42.85^ab^	531.26^c^	391.82^c^	0.32^a^
	T_7_	17.00^d^	8.72^d^	37.98^d^	287.03^f^	191.57^g^	0.25^a^
	Mean	28.36	18.39	41.86	488.78	352.54	0.31
	Grand mean	58.86	16.49	40.15	975.01	693.91	0.31
LSD (p ≤ 0.01)	Variety	0.55	4.24	6.43	31.96	21.04	0.06
	Treatment	0.89	0.67	1.04	25.59	27.56	0.24
	V × T	0.87	0.29	0.13	23.60	17.17	0.02

Within column means followed by the same superscripts are not significantly different at p ≤ 0.01

T_1_: Pendimethalin at 1.5 l ha^−1^, T_2_: Pendimethalin at 1.5 l ha^−1^ + one hand weeding at 6 WAS, T_3_: Rice straw mulch, T_4_: Rice straw mulch + one hand weeding at 6 WAS, T_5_: Two hand weeding at 3 and 6 WAS, T_6_: Weed free check (positive control), T_7_: Weedy check (negative control)

In conclusion, rice straw mulch at 0.1 m depth + one hand weeding at 6 week after sowing increased growth and yield of the two varieties when compared to other weed control methods. The enhancement of crop productivity in this treatment could be attributed to its positive influence on LAI, NAR, RGR and CGR.
